# Clinician Staffing and Quality of Care in US Health Centers

**DOI:** 10.1001/jamanetworkopen.2024.40140

**Published:** 2024-10-22

**Authors:** Q. Wilton Sun, Howard P. Forman, Logan Stern, Benjamin J. Oldfield

**Affiliations:** 1 Yale School of Medicine, New Haven, Connecticut; 2 Department of Radiology and Biological Imaging, Yale School of Medicine, New Haven, Connecticut; 3 Department of Health Policy and Management, Yale School of Public Health, New Haven, Connecticut; 4 Yale School of Management, New Haven, Connecticut; 5 Fair Haven Community Health Care, New Haven, Connecticut; 6 Department of Medicine, Yale School of Medicine, New Haven, Connecticut; 7 Department of Pediatrics, Yale School of Medicine, New Haven, Connecticut

## Abstract

**Importance:**

Health centers are vital primary care safety nets for underserved populations, but optimal clinician staffing associated with quality care is unclear. Understanding the association of clinician staffing patterns with quality of care may inform care delivery, scope-of-practice policy, and resource allocation.

**Objective:**

To describe the association of clinician staffing models and ratios with quality-of-care metrics in health centers.

**Design, Setting, and Participants:**

This cross-sectional study of US health centers used data from the 2022 Health Resources and Services Administration Uniform Data System (UDS).

**Exposure:**

Clinician staffing ratios, expressed as the fraction of full-time equivalents (FTEs) per 1000 visits of physicians, advanced practice registered nurses (APRNs), and physician associates (PAs) to total clinician FTEs at each health center.

**Main outcomes and measures:**

Percentage of eligible patients receiving the corresponding service or outcome for 14 individual clinical quality metrics collected by the UDS, including infant immunizations; screening for cancer, depression, tobacco use, hypertension, HIV, and glycated hemoglobin levels; weight and body mass index (BMI) assessment; and appropriate statin, aspirin, and/or antiplatelet therapy.

**Results:**

This analysis of 791 health centers serving 16 114 842 patients (56.6% female) identified 5 clinician staffing models: balanced (similar FTEs of physicians, APRNs, and PAs; 152 [19.2%] of health centers), higher FTEs of APRNs than physicians (174 [22.0%]), higher FTEs of physicians than APRNs (160 [20.2%]), approximately equal FTEs of physicians and APRNs (263 [33.2%]), and large scale (42 [5.3%]). Adjusted linear models showed positive associations between physician FTEs per 1000 visits and cervical (β, 14.9; 95% CI, 3.1-26.7), breast (β, 15.7; 95% CI, 3.2-28.1), and colorectal (β, 18.3; 95% CI, 6.0-30.6) cancer screening. Generalized additive models showed nonlinear positive associations beginning at a physician FTE ratio of 0.45 (95% CI, 0.02-6.22) for infant vaccinations, 0.39 (95% CI, 0.05-2.21) for cervical cancer screening, 0.39 (95% CI, 0.02-1.67) for breast cancer screening, 0.47 (95% CI, 0.00-5.76) for HIV testing, and 0.70 (95% CI, 0.18-19.96) for depression in remission; APRN FTE ratio of 0.45 (95% CI, 0.17-7.46) for adult BMI assessment; and PA FTE ratio of 0.16 (95% CI, 0.11-3.88) for infant vaccinations. Staffing models were not associated with 7 of the 14 metrics analyzed.

**Conclusions and Relevance:**

In this cross-sectional study of health centers, physician FTE ratio was associated with higher performance in cancer screening, infant vaccinations, and HIV testing; APRN FTE ratio was associated with higher performance in preventative health assessments; and PA FTE ratio was associated with higher performance in infant vaccination. These findings suggest that targeted staffing strategies may be associated with quality of care in certain domains and that tailored approaches to health center staffing based on community-specific needs are warranted.

## Key Points

**Question:**

Is there an association between clinician staffing (physicians and advanced practice practitioners) and quality-of-care metrics in health centers in the US?

**Findings:**

In this cross-sectional study of 791 US health centers, 5 distinct clinician staffing ratio models were identified, and models emphasizing physicians, advanced practice registered nurses, and physician associates were positively associated with distinct sets of individual quality metrics. Staffing models were not associated with 7 of 14 metrics analyzed.

**Meaning:**

Clinician staffing may be associated with certain aspects of care quality, underscoring the importance of strategic, tailored staffing to optimize primary care delivery.

## Teaser

This cross-sectional study examines whether predominant clinician staffing patterns in US health centers are associated with specific metrics for quality of clinical care.

## Introduction

Health centers, sometimes referred to as federally qualified health centers, are community-based primary care safety net institutions; these centers served 31.5 million patients across urban and rural settings in the US in 2022.^1^ Beyond the foundational dedication of health centers to medically underserved populations,^2^ utilization of these centers is associated with decreased rates of emergency department visits and hospital admissions.^3,4,5,6^ Expansion of Medicaid has increased health center patient capacity^7^ and been associated with improved chronic disease management and outcomes.^8,9^

Due to long-standing shortages of primary care physicians,^10^ increased demand for health center services^7^ precipitated by Medicaid expansion, evolving health center patient populations^11^ with increasing chronic disease multimorbidity,^12^ and COVID-19 disruptions in health center care delivery,^13^ maintaining an optimal clinician workforce remains a significant operational challenge for health centers. Concurrently, the growing advanced practice practitioner (APP) workforce may potentially fill the void of primary care physicians.^14,15^ Use of nurse practitioners, certified nurse midwives, and physician associates (PAs) (all considered APPs) in health centers is greater than that in other office-based practices,^16^ is cost-effective,^17^ and may be influenced by scope-of-practice laws, Medicaid expansion status, and rural designation.^18,19,20^ The evolving paradigm in the primary care workforce necessitates an understanding of the influence of clinician staffing models on patient care.

Few studies have addressed quality of care and patient outcomes in the context of health center clinician staffing. A comparative study of physicians and APPs from 2006 to 2010 found greater odds of smoking cessation, health education, and counseling services provided by APPs than physicians.^21^ Another study reported improvements in the proportion of patients with well-controlled hypertension and diabetes associated with increases in physician or APP full-time equivalent (FTE).^22^ Additionally, physicians and APPs do not differ in visit time with patients.^23^ While these studies explored a variety of metrics, they did not include metrics related to health maintenance milestones, such as vaccinations or cancer screenings, or extensively characterized nonlinear relationships between clinician staffing and quality of care. Using linear and nonlinear modeling approaches, we sought to (1) characterize predominant clinician staffing patterns among health centers, (2) identify specific quality metrics associated with variations in clinician staffing, and (3) establish clinician staffing ratio thresholds for enhancing care quality.

## Methods

### Data Source

We performed a cross-sectional study of health centers using the 2022 Health Resources and Services Administration Uniform Data System (UDS). The UDS releases annual data on patient population, organizational characteristics, and quality of care reported at the health center level. Staffing data are deemed proprietary^24,25^ and withheld from release^26^ if less than 10 years old and designated by health centers as confidential commercial information. Thus, the study sample included all health centers that had consented to release staffing data to the UDS (characteristics of nonconsenting health centers are in eTable 1 in Supplement 1). This study was not considered human participant research by the Yale School of Medicine human investigation committee and followed the Strengthening the Reporting of Observational Studies in Epidemiology (STROBE) reporting guideline.^27^

### Outcome Measures

Quality of care in health centers was assessed through 14 UDS-collected individual clinical quality metrics,^28^ which include rates of appropriate infant immunizations, cervical cancer screening, breast cancer screening, child and adolescent weight assessment and counseling, adult body mass index (BMI) assessment, tobacco use screening, statin therapy, aspirin and/or antiplatelet therapy, colorectal cancer screening, HIV testing, depression screening, depression remission at 12 months, adequately controlled hypertension, and poorly controlled glycated hemoglobin. All are expressed as percentage of eligible patients receiving the corresponding appropriate service or outcome (a detailed description of the metrics is given by the UDS).^28^

### Independent Measures and Covariates

Staffing models were represented as FTEs of physicians (primary care and specialty), advanced practice registered nurses (APRNs; including nurse practitioners and certified nurse midwives), and PAs per 1000 visits per year and as staffing ratios (hereafter, *staffing ratio* refers to a health center–specific variable that refers to the ratio of a subgroup of clinician FTEs to all clinician [physician, APRN, and PA] FTEs per 1000 visits at that health center). Analyses controlled for the following health center–level covariates most plausibly related to staffing opportunities and services rendered: rural or urban designation and health center–attributed patient population demographics (race and ethnicity, limited English proficiency, sex, income, and uninsured status). Patient race and ethnicity were ascertained by self-report; categories were non-Hispanic Black, Hispanic or Latinx, non-Hispanic White, and other (American Indian or Alaska Native, Asian, Hawaiian or Pacific Islander, or multiracial).

### Statistical Analysis

As the unit of analysis was at the health center level, we first elucidated the most salient clinician staffing models using a *k*-means clustering algorithm set at a *k* of 5 clusters. This unsupervised learning technique autonomously categorized health centers into distinct clusters that naturally emerged from the data based on similar staffing configurations, minimizing the variance in characteristics within clusters while maximizing the differences between clusters. We presented the staffing models of physician, APRN, and PA and the patient population characteristics of each cluster with silhouette scores, which quantify distinction between clusters on a scale from −1 to 1, where higher scores indicate more well-distinguished clusters.

Linear and nonlinear approaches were used to model the association between clinician staffing and quality of care, acknowledging the potential of nonlinear associations in which quality may improve, plateau, or decline along staffing ratio spectrums. Multivariate linear regression models assessed clinician FTEs per 1000 visits per year and individual quality-of-care metrics, reported as regression β coefficients with 95% CIs. We included models unadjusted and adjusted for covariates and further adjusted for 2-way and 3-way interaction effects between physicians, APRNs, and PAs to account for interdisciplinary dynamic effects potentially inherent among clinician types in team-based care settings.

Generalized additive models (GAMs) assessed the presence of nonlinear associations between FTE ratios and individual quality metrics, controlling for covariates. General additive models apply smooth functions enabling the model to conform to the true trends of the data rather than imposing a predetermined curve configuration. If significant nonlinear associations were detected, we calculated the minimum FTE ratio at which the corresponding quality metric increased by determining the point at which the curve and lower 95% CI bound crossed 0 on the y-axis. All analyses were performed in R Studio, version 4.2.1 (R Project for Statistical Computing) at a significance of α = .05, with 2-sided *P* values adjusted for multiple comparisons using the Benjamini-Hochberg false discovery procedure.

## Results

A total of 791 health centers (57.7% of all 1370 health centers in 2022) were included, serving 16 114 842 patients (56.6% female; 43.4% male). Overall, 16.2% of patients were Black; 34.8%, Hispanic or Latinx; 36.7%, White; and 12.3%, other race and ethnicity. Five predominant clinician staffing models were identified (Table 1): 152 health centers (19.2%) had a roughly balanced FTE of physicians, APRNs, and PAs; 174 health centers (22.0%) had a higher FTE of APRNs than physicians; 160 health centers (20.2%) had a higher FTE of physicians than APRNs; and 263 health centers (33.2%) had roughly equal FTEs of physicians and APRNs. Forty-two health centers (5.3%) were distinguished by substantial staffing sizes, with a mean (SD) of 58.3 (22.7) physician, 32.4 (15.8) APRN, and 11.2 (14.0) PA FTEs (210%-470% larger than the respective workforces in all other models). Health centers from the high-APRN, high-physician, and predominantly physician and APRN models had a minimal FTE of PAs. The models yielded a mean (SD) silhouette score of 0.34 (0.18), suggesting moderate distinction. Health centers in the high-APRN group tended to be in rural service areas and served a greater proportion of uninsured patients, while large-scale health centers served a greater proportion of racial and ethnic minority patients. Nonconsenting health centers did not differ greatly from consenting health centers in geographic spread and patient demographics (eTable 1 in Supplement 1).

**Table 1.** Characteristics of 791 US Health Centers by Staffing Cluster, 2022

Characteristic	Health centers				
	Balanced (n = 152)	High APRN (n = 174)	High physician (n = 160)	Predominantly physician and APRN (n = 263)	Large scale (n = 42)
**Staffing characteristics**					
Physicians					
FTEs, mean (SD)	5.6 (5.8)	2.2 (3.4)	12.4 (9.6)	7.3 (6.0)	58.3 (22.7)
%^a^	33.9	19.1	63.9	39.7	57.2
APRNs					
FTEs, mean (SD)	5.6 (5.5)	8.8 (11.8)	5.3 (4.9)	9.8 (8.2)	32.4 (15.8)
%^a^	33.9	76.5	27.3	53.3	31.8
PAs					
FTEs, mean (SD)	5.3 (4.2)	0.5 (1.1)	1.7 (2.7)	1.3 (2.4)	11.2 (14.0)
%^a^	32.1	4.3	8.8	7.1	11.0
RNs					
RN FTEs, mean (SD)	9.5 (10.5)	11.2 (22.9)	14.4 (16.4)	13.7 (14.8)	67.5 (49.3)
Support staff FTEs, mean (SD)^b^	22.5 (23.9)	10.3 (12.2)	25.4 (27.2)	22.4 (22.7)	139.0 (101.0)
Rural service area, No. (%)	79 (52.0)	94 (54.0)	45 (28.1)	104 (39.5)	7 (16.7)
**Patient characteristics, No. (%)**					
Total patients, No.	2 441 847	1 907 694	3 079 632	4 857 770	3 827 899
Sex					
Female	1 360 109 (55.7)	1 051 139 (55.1)	1 770 788 (57.5)	2 710 636 (55.8)	2 224 009 (58.1)
Male	1 081 738 (44.3)	856 555 (44.9)	1 308 844 (42.5)	2 147 134 (44.2)	1 603 890 (41.9)
Age, y					
0-17	629 997 (25.8)	473 108 (24.8)	963 925 (31.3)	1 345 602 (27.7)	1 151 432 (30.1)
18-64	1 496 852 (61.3)	1 222 832 (64.1)	1 761 550 (57.2)	2 934 093 (60.4)	2 185 730 (57.1)
≥65	314 998 (12.9)	211 754 (11.1)	354 157 (11.5)	578 075 (11.9)	490 737 (12.8)
Race and ethnicity					
Black^c^	214 883 (8.8)	356 739 (18.7)	520 458 (16.9)	971 554 (20.0)	547 390 (14.3)
Hispanic or Latinx	913 251 (37.4)	351 016 (18.4)	1 259 569 (40.9)	1 321 313 (27.2)	1 760 834 (46.0)
White^c^	1 030 459 (42.2)	1 018 709 (53.4)	834 580 (27.1)	2 035 406 (41.9)	1 002 910 (26.2)
Other^d^	283 254 (11.6)	181 230 (9.5)	465 025 (15.1)	529 497 (10.9)	516 765 (13.5)
Limited English proficiency	510 346 (20.9)	286 154 (15.0)	945 447 (30.7)	850 110 (17.5)	1 221 100 (31.9)
Uninsured	493 253 (20.9)	507 447 (26.6)	665 201 (21.6)	966 696 (19.9)	581 841 (15.2)
Income <100% FPL	925 460 (37.9)	858 462 (45.0)	1 407 392 (45.7)	2 151 992 (44.3)	1 527 332 (39.9)

Abbreviations: APRN, advanced practice registered nurse; FPL, federal poverty level; FTE, full-time equivalent; physician associate; RN, registered nurse.

^a^ Among all clinicians (physicians, APRNs, and PAs), calculated using the given mean FTE for each cluster.

^b^ Includes medical and nursing assistants.

^c^ Only includes patients identifying as not Hispanic or Latinx.

^d^ Patients self-reporting as American Indian or Alaska Native, Asian, Hawaiian or Pacific Islander, or multiracial were grouped together due to small percentages.

Multivariate linear models showed that staffing models with higher physician FTEs (large scale and high physician) were positively associated with better health center performance in quality metrics related to cancer screenings and HIV testing. Physician FTEs per 1000 visits per year were positively associated with rates of cervical cancer screening (β, 15.9; 95% CI, 4.3-27.5; *P* = .01), colorectal cancer screening (β, 16.1; 95% CI, 4.5-27.8; *P* = .01), and HIV testing (β, 49.8; 95% CI, 32.6-67.1; *P* < .001) (Table 2). The associations with cervical cancer screening (β, 14.9; 95% CI, 3.1-26.7; *P* = .04) and colorectal cancer screening (β, 18.3; 95% CI, 6.0-30.6; *P* = .002) remained when adjusted for covariates. Additionally, physician FTEs per 1000 visits were positively associated with rates of breast cancer screening (β, 15.7; 95% CI, 3.2-28.1; *P* = .04) in the adjusted model. The APRN and PA FTEs per 1000 visits were not positively associated with any quality metrics in the linear model. These findings were generally upheld in sensitivity analyses accounting for interaction effects between clinician types (eTables 2 and 3 in Supplement 1). Child and adolescent BMI assessment, tobacco use screening, statin prescribing, aspirin and/or antiplatelet prescribing, depression, hypertension, and diabetes outcomes were independent of clinician FTEs per 1000 visits in linear models.

**Table 2.** Linear Models of Clinician FTEs per 1000 Visits and Individual Quality Metrics in 791 US Health Centers, 2022

Quality metric^a^	β (95% CI)					
	Physician FTEs per 1000 visits		APRN FTEs per 1000 visits		PA FTEs per 1000 visits	
	Unadjusted	Adjusted^b^	Unadjusted	Adjusted^b^	Unadjusted	Adjusted^b^
Infant vaccination	14.8 (−0.2 to 29.8)	12.6 (−3.7 to 28.9)	−17.2 (−27.6 to −6.9)^c^	−8.6 (−19.9 to 2.7)	−20.7 (−37.3 to −4.0)	−8.6 (−28.9 to 6.8)
Cervical cancer screening	15.9 (4.3 to 27.5)^c^	14.9 (3.1 to 26.7)^c^	−16.3 (−31.2 to −5.4)^c^	−5.8 (−14.0 to −11.4)	−18.3 (−31.1 to −5.4)	−1.5 (−14.5 to 11.4)
Breast cancer screening	12.0 (0.1 to 23.9)	15.7 (3.2 to 28.1)^c^	−12.8 (−21.0 to −4.5)^c^	−9.0 (−17.6 to −0.4)	−9.4 (−22.6 to 3.9)	−0.7 (−14.3 to 13.0)
Child and adolescent BMI assessment	−7.1(−24.5 to −10.3)	−6.7 (−26.2 to 12.8)	−10.9 (−23.1 to −1.1)	−7.8 (−21.3 to 5.6)	−61.2 (−80.6 to −41.9)	−53.3 (−74.7 to −32.0)^c^
Adult BMI assessment	−45.1 (−61.8 to −28.4)^c^	−41.5 (−60.2 to −22.7)^c^	−1.5 (−13.0 to −10.1)	−8.7 (−21.7 to 4.2)	−37.0 (−55.5 to −18.4)	−32.2 (−52.8 to −11.7)^c^
Tobacco screening	−14.4 (−25.1 to −3.6)^c^	−10.7 (−22.6 to 1.3)	−7.9 (−15.4 to −0.4)^c^	−10.6 (−18.8 to −2.3)^c^	−4.3 (−16.3 to 7.7)	−2.0 (−15.1 to 11.1)
Statin therapy	3.2 (−3.4 to 9.7)	2.9 (−4.1 to 9.9)	−0.5 (−5.1 to −4.0)	1.6 (−3.3 to 6.5)	−12.0 (−19.3 to −4.7)	−6.7 (−14.4 to 1.0)
Aspirin and/or antiplatelet therapy	0.3 (−8.9 to 9.5)	1.1 (−9.0 to 11.2)	−6.1 (−12.4 to 0.3)	−3.4 (−10.4 to 3.6)	−2.1 (−12.4 to 8.1)	−1.0 (−12.1 to 10.0)
Colorectal cancer screening	16.1 (4.5 to 27.8)^c^	18.3 (6.0 to 30.6)^c^	−8.8 (−16.9 to −0.7)^c^	−3.7 (−12.2 to 4.9)	5.4 (−7.6 to 18.4)	10.9 (−2.6 to 24.4)
HIV testing	49.8 (32.6 to 67.1)^c^	16.8 (1.5 to 32.0)	0.6 (−11.3 to 12.6)	7.9 (−2.6 to 18.4)	−37.5 (−56.7 to −18.3)	−21.0 (−37.7 to −4.3)^c^
Depression screening	−9.6 (−24.0 to 4.9)	−4.0 (−20.2 to 12.1)	4.5 (−5.5 to 14.5)	6.0 (−5.2 to 17.2)	−22.8 (−38.9 to −6.7)	−18.7 (−36.5 to −1.0)
Depression in remission	6.3 (−5.9 to 18.5)	1.2 (−11.8 to 14.3)	−1.3 (−9.8 to 7.1)	−0.1 (−9.1 to 9.0)	−21.1 (−34.8 to −7.6)	−9.7 (−24.1 to 4.6)
Hypertension controlled	−10.2 (−17.0 to −3.4)	−6.1 (−13.2 to 1.0)	−5.5 (−10.2 to −0.7)	−2.9 (−7.8 to 2.0)	−2.0 (−9.6 to 5.6)	1.9 (−5.9 to 9.7)
HbA_1c_ level >9%	1.9 (−4.8 to 8.7)	1.4 (−5.5 to 8.4)	1.9 (−2.8 to 6.5)	3.6 (−1.3 to 8.4)	−2.8 (−10.3 to 4.7)	−1.2 (−8.8 to 6.5)

Abbreviations: APRN, advanced practice registered nurse; BMI, body mass index; FTE, full-time equivalent; HbA_1c_, glycated hemoglobin; PA, physician associate.

SI conversion: to convert the percentage of total HbA_1c_ to the proportion of total HbA_1c_, multiply by 0.01.

^a^ Measurements of individual quality metrics are defined by the UDS.^28^

^b^ Accounts for rural or urban designation, race and ethnicity, limited English proficiency, sex, income, and uninsured status.

^c^ *P* < .05.

**Figure 1.**
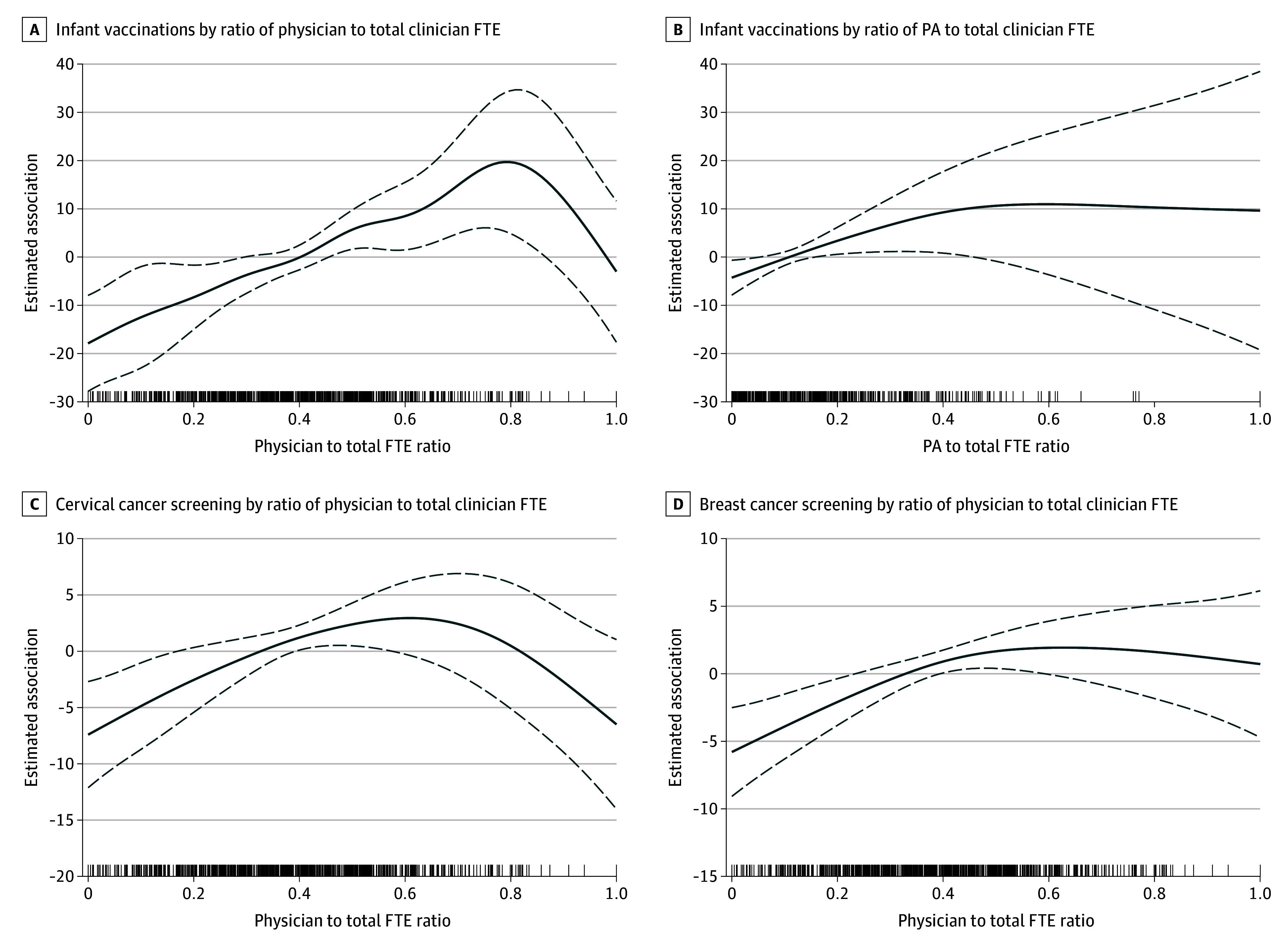
Generalized Additive Models of Associations Between Full-Time Equivalent (FTE) Staffing Ratios per 1000 Visits and Infant Vaccinations, Breast Cancer Screening, and Cervical Cancer Screening in 791 US Health Centers The y-axes quantify the estimated association of the corresponding FTE per 1000 visits, expressed as a ratio of total physician, advanced practice registered nurse, and physician associate FTEs per 1000 visits, with the specified quality metric. Solid lines represent the smooth function, which approximates the actual shape of data rather than imposing a predetermined fit; dotted lines, 95% CIs; lines above the x-axis, data points corresponding to each of the 791 health centers showing the exact distribution of their specific staffing ratios. PA indicates physician associate.

**Figure 2.**
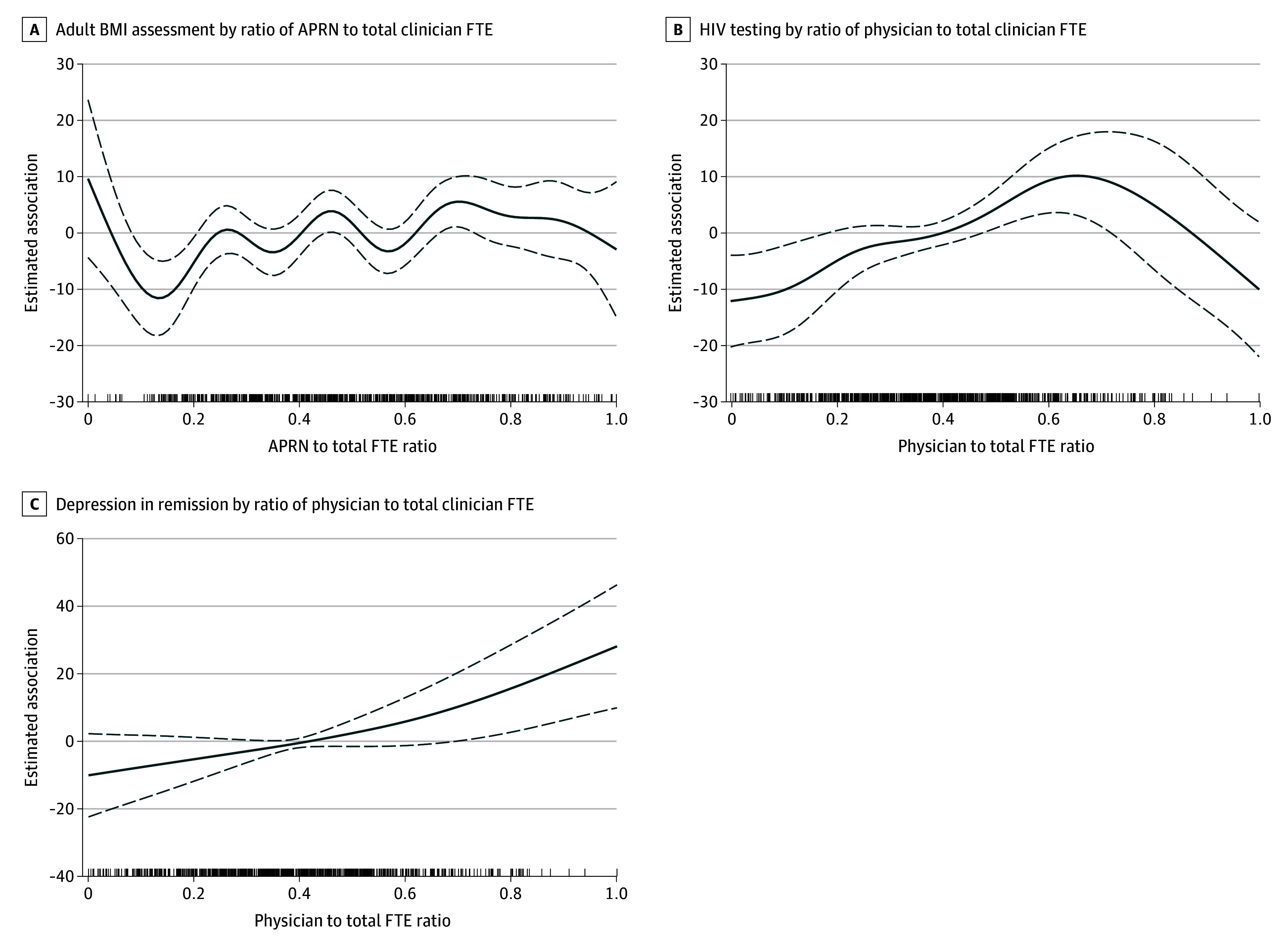
Generalized Additive Models for Associations Between Full-Time Equivalent (FTE) Staffing Ratios per 1000 Visits and Adult Body Mass Index (BMI) Assessment, HIV Testing, and Depression in Remission in 791 US Health Centers The y-axes quantify the estimated association of the corresponding FTE per 1000 visits, expressed as a ratio of total physician, advanced practice registered nurse (APRN), and physician associate FTEs per 1000 visits, with the specified quality metric. Solid lines represent the smooth function, which approximates the actual shape of data rather than imposing a predetermined fit; dotted lines, 95% CIs; lines above the x-axis, data points corresponding to each of the 791 health centers showing the exact distribution of their specific staffing ratios.

The GAM analyses identified positive, nonlinear associations between staffing ratios and individual quality metrics when controlling for covariates. Figure 1 and Figure 2 illustrate estimated associations as functions of ratios of FTEs per 1000 visits for which statistically significant associations existed. Figure 1A and B illustrate a positive association with the rate of infant vaccinations beginning at a minimum FTE ratio of 0.45 (95% CI, 0.02-6.22; *P* = .04) for physicians and 0.16 (95% CI, 0.11-3.88; *P* = .04) for PAs, respectively. These ratios were roughly equivalent to the predominantly physician-APRN staffing model. The minimum physician FTE count at which this association became positive was 9.25 (1.31; 95% CI, 0.10-2.52), but there was no association when adjusted for multiple comparisons (eFigure 1 in Supplement 1). The GAMs suggested no association between PA FTE counts and rates of infant vaccinations, with no minimum point of change (eFigure 2 in Supplement 1).

Figure 1C and D illustrate a positive association with rates of cervical and breast cancer screening beginning at a minimum physician FTE ratio of 0.39 (95% CI, 0.05-2.21; *P* = .02) and 0.39 (95% CI, 0.02-1.67; *P* = .03), respectively, roughly equivalent to the balanced staffing model. The minimum physician FTE count at which this association became positive was 6.61 for cervical cancer screening (1.14; 95% CI, 0.01-2.27; *P* = .002) and 7.93 for breast cancer screening (2.05; 95% CI, 0.56-3.54; *P* = .006) (eFigures 3 and 4 in Supplement 1). The GAMs identified no association between physician FTE ratios and colorectal cancer screening, but an association between physician FTE counts and colorectal cancer screening beginning at an FTE count of 7.93 (1.91; 95% CI, 0.47-3.34; *P* = .02) was revealed (eFigure 5 in Supplement 1).

Figure 2A illustrates a positive association with rates of adult BMI assessment and counseling beginning at a minimum APRN FTE ratio of 0.45 (95% CI, 0.17-7.46; *P* = .01), roughly equivalent to the predominantly physician-APRN staffing model. The GAMs identified a positive linear association between APRN FTE counts and rates of adult BMI assessment, with no minimum point of change (eFigure 6 in Supplement 1). Figure 2B illustrates a positive association with rates of HIV testing beginning at a minimum physician FTE ratio of 0.47 (95% CI, 0.00-5.76; *P* = .007) or 14.53 FTEs (1.40; 95% CI, 0.07-2.72; *P* = .04) (eFigure 7 in Supplement 1), roughly equivalent to ratios in the predominantly physician-APRN staffing model. The GAMs revealed no association between APRN FTE ratios and HIV testing, but there was an association between APRN FTE counts and HIV testing beginning at 8.81 FTEs (1.46; 95% CI, 0.10-2.81; *P* = .02) (eFigure 8 in Supplement 1). Figure 2C illustrates a positive association with rates of depression in remission beginning at a minimum physician FTE ratio of 0.70 (95% CI, 0.18-19.96; *P* = .04), roughly equivalent to the high physician staffing model. The GAMs did not reveal an association between physician FTE counts and rates of depression in remission (eFigure 9 in Supplement 1). The GAMs did not reveal associations between staffing ratios and any of the other quality metrics.

## Discussion

In this cross-sectional study of 791 health centers in 2022, 5 predominant clinician staffing configurations were identified (balanced physician, APRN, and PA; high APRN; high physician; predominantly physician and APRN; and large scale). Multivariate linear models identified positive linear associations between physician staffing models and quality metrics related to cancer screenings and HIV testing. The GAMs identified positive nonlinear associations between physician staffing ratios and infant vaccinations, cancer screening, and HIV testing; APRN staffing ratios and adult BMI assessment; and PA staffing ratios and infant vaccinations, with specific staffing ratios for positive associations.

While many researchers have postulated that primary care physicians and APPs are interchangeable,^15,19,21,22^ our findings suggest more nuanced, complementary roles. The UDS does not delineate specific practitioners responsible for individual patient visits, but a previous study observed greater rates of cancer screening among Medicare beneficiaries receiving care from primary care physicians.^29^ Similarly, APRNs and PAs are more likely to provide health education and counseling services.^21,30^ These correlations have several implications. First, although health maintenance activities such as cancer screening are cornerstones of primary care delivery,^31,32^ few health centers meet cancer screening benchmarks.^33^ We found that at least 6 to 8 physician FTEs, or a physician ratio of FTEs per 1000 visits of 0.39, were positively associated with cancer screening, indicating that strategic calibration of staffing may in part be an important consideration in improving cancer screening among underserved populations. Second, increases in APRN FTE ratios may be associated with health center–wide adoption of screening and health promotion activities. We found that APRN staffing ratios of at least 0.45 were positively associated with increased rates of adult BMI assessment and counseling. Third, team-based care models have often been proposed to address primary care service shortages.^34,35,36,37^ While multidisciplinary teams are not new in health centers,^38,39^ specific role clarification may equip various clinicians to excel in specific domains^34^ and enhance team performance. Furthermore, given funding uncertainties of health centers,^40^ our findings of specific staffing thresholds associated with quality provide a framework for strategic optimization of clinician recruitment without compromising resources for other essential services.

Importantly, neither multivariate linear models nor GAMs identified associations with 7 of the 14 quality metrics analyzed in this study, including metrics related to hypertension or diabetes control and appropriate statin or antiplatelet therapies. While specific roles in patient care and clinician strengths may vary between physicians and APPs, quality of care is often equivalent in hypertension control and quality of prescribing,^21,41,42^ suggesting that quality improvement does not change significantly based on staffing configurations alone, with a lack of superior staffing configuration yielding universally optimal outcomes. Furthermore, the associations identified in this study may be reflective of not only the clinicians’ attributes but broader organization-wide factors including leadership and governance,^43^ institutional policies, quality improvement priority setting through governing boards and patient advisory councils,^44^ timely access to care, and health information technology strategies to address gaps in care^45^ while reducing practitioner burnout.^46^

Health centers use diverse staffing configurations, which may be partly influenced by community-specific needs as well as legislative environments such as scope of practice. Notably, 3 of the 5 clusters (high APRN, high physician, and predominantly physician and APRN) included a minimal proportion of PAs, potentially related to limited job postings for PAs in primary care,^47^ dependency on physicians rather than APRNs as supervisors,^48^ and decreasing federal support promoting use of PAs in primary care.^49^ Relatedly, hiring APRNs may be more cost-effective for health centers,^17,22,50^ but their use may be influenced in states where scope-of-practice laws limit full practice authority.^18^ It is essential that scope-of-practice laws be flexible and adaptable to meet the diverse needs of different health care settings, especially within health centers, both between and within states.

This study has additional policy and practice implications. As value-based reimbursement models become incorporated into health centers,^51,52^ consideration of efficient clinician workforce configurations may inform hiring decisions focused on care quality. Additionally, more predictable funding structures^40^ and reimbursement models that better accommodate team-based care and care coordination^53^ may enhance workforce planning and stability. Furthermore, as loan forgiveness programs have not increased physician density in shortage areas,^54^ such programs could instead target placement of practitioners in regions seeking to improve quality in specific domains.

### Limitations

There are several study limitations. First, the design of this analysis was associative, and causal interpretations cannot be inferred. Analyses were confined to a single year in health centers that had consented to release of workforce data through the UDS. Workforce data were only reported as FTEs and may not capture the full complexities of job sharing, part-time work, or overtime hours. Reported FTEs imply total equivalents worked, not necessarily patient-facing time; this may have overestimated the clinical time of physicians who were more likely to have administrative and supervisory duties in addition to patient care duties. Factors such as the burden of medical complexity of patients, the prevalence of health-related social drivers, the degree to which quality improvement initiatives (eg, in the form of reminders, pathways, or other such system changes) have been undertaken at health centers, and support personnel to execute these initiatives were not available. Additionally, data were reported at the health center level, so we were unable to assess direct clinical impact. Nevertheless, this study provides important insights into staffing contributions to care quality across a comprehensive, standardized set of metrics in health centers, which are ideal settings to examine clinician staffing and clinical quality.

## Conclusions

This cross-sectional study characterized clinician staffing patterns within health centers, revealing differentiated associations between physicians, APRNs, and PAs with individual clinical quality care metrics. Physician staffing was associated with increased cancer screening, infant vaccinations, and HIV testing; APRN staffing was associated with adult BMI assessment and counseling; and PA staffing was associated with infant vaccinations. These findings suggest that strategic staffing may be associated with optimization of primary care delivery in health centers, with adoption of value-based reimbursement models, readjustment of programs in areas with health professional shortages, and strengthened organizational leadership as key factors to incentivize quality-driven changes to workforce configurations.

## Notes

Supplement 1. **eTable 1.** Patient Population Characteristics of Nonconsenting Health Centers, 2022 **eTable 2.** Multivariate Linear Models for Clinician FTE and Individual Quality Metrics With Interaction Terms Between Clinician Types **eTable 3.** Multivariate Linear Models for Clinician FTE per 1000 Visits and Individual Quality Metrics With Interaction Terms Between Clinician Types **eFigure 1.** Generalized Additive Models of Physician FTE and Rates of Infant Vaccinations **eFigure 2.** Generalized Additive Models of PA FTE and Rates of Infant Vaccinations **eFigure 3.** Generalized Additive Models of Physician FTE and Rates of Cervical Cancer Screening **eFigure 4.** Generalized Additive Models of Physician FTE and Rates of Breast Cancer Screening **eFigure 5.** Generalized Additive Models of Physician FTE and Rates of Colorectal Cancer Screening **eFigure 6.** Generalized Additive Models of APRN FTE and Rates of Adult BMI Assessment and Counseling **eFigure 7.** Generalized Additive Models of Physician FTE and Rates of HIV Testing **eFigure 8.** Generalized Additive Models of APRN FTE and Rates of HIV Testing **eFigure 9.** Generalized Additive Models of Physician FTE and Rates of Depression in Remission

Supplement 2.
Data Sharing Statement
