# Supplementary material for: Clinician Staffing and Quality of Care in US Health Centers
Source: JAMA Netw Open. 2024 Oct 22;7(10):e2440140. doi: 10.1001/jamanetworkopen.2024.40140 (PMC11581487; doi:10.1001/jamanetworkopen.2024.40140)
Supplement: Supplement 1. — eTable 1. Patient Population Characteristics of Nonconsenting Health Centers, 2022 eTable 2. Multivariate Linear Models for Clinician FTE and Individual Quality Metrics With Interaction Terms Between Clinician Types eTable 3. Multivariate Linear Models for Clinician FTE per 1000 Visits and Individual Quality Metrics With Interaction Terms Between Clinician Types eFigure 1. Generalized Additive Models of Physician FTE and Rates of Infant Vaccinations eFigure 2. Generalized Additive Models of PA FTE and Rates of Infant Vaccinations eFigure 3. Generalized Additive Models of Physician FTE and Rates of Cervical Cancer Screening eFigure 4. Generalized Additive Models of Physician FTE and Rates of Breast Cancer Screening eFigure 5. Generalized Additive Models of Physician FTE and Rates of Colorectal Cancer Screening eFigure 6. Generalized Additive Models of APRN FTE and Rates of Adult BMI Assessment and Counseling eFigure 7. Generalized Additive Models of Physician FTE and Rates of HIV Testing eFigure 8. Generalized Additive Models of APRN FTE and Rates of HIV Testing eFigure 9. Generalized Additive Models of Physician FTE and Rates of Depression in Remission [file jamanetwopen-e2440140-s001.pdf]

Supplementary Online Content

Sun QW, Forman HP, Stern L, Oldfield BJ. Clinician staffing and quality of care in US health centers. *JAMA Netw Open*. 2024;7(10):e2440140. doi:10.1001/jamanetworkopen.2024.40140

eTable 1. Patient Population Characteristics of Nonconsenting Health Centers, 2022
eTable 2. Multivariate Linear Models for Clinician FTE and Individual Quality Metrics With Interaction Terms Between Clinician Types
eTable 3. Multivariate Linear Models for Clinician FTE per 1000 Visits and Individual Quality Metrics With Interaction Terms Between Clinician Types
eFigure 1. Generalized Additive Models of Physician FTE and Rates of Infant Vaccinations
eFigure 2. Generalized Additive Models of PA FTE and Rates of Infant Vaccinations
eFigure 3. Generalized Additive Models of Physician FTE and Rates of Cervical Cancer Screening
eFigure 4. Generalized Additive Models of Physician FTE and Rates of Breast Cancer Screening
eFigure 5. Generalized Additive Models of Physician FTE and Rates of Colorectal Cancer Screening
eFigure 6. Generalized Additive Models of APRN FTE and Rates of Adult BMI Assessment and Counseling
eFigure 7. Generalized Additive Models of Physician FTE and Rates of HIV Testing
eFigure 8. Generalized Additive Models of APRN FTE and Rates of HIV Testing
eFigure 9. Generalized Additive Models of Physician FTE and Rates of Depression in Remission

This supplementary material has been provided by the authors to give readers additional information about their work.

© 2024 Sun QW et al. *JAMA Network Open*.

eTable 1. Patient population characteristics of nonconsenting health centers, 2022.

Health Centers, No. [a]	579
Rural service area, No.	237
Total patients, No.	14,402,434
Sex, No.	
Female	8,308,690
Male	6,093,744
Age, No.	
0-17	4,190,776
18-64	8,544,941
≥65	1,638,622
Race and ethnicity, No.	
Black [b]	2,416,249
Hispanic/Latinx	5,432,520
White [b]	4,503,651
Other [c]	2,050,014
Limited English proficiency, No.	3,835,666
Uninsured, No.	2,671,006
Income < 100% FPL, No.	6,618,953

[a] Includes health centers (42.3% of all health centers) that did not consent to release of staffing data through UDS.
[b] Only includes patients identifying as non-Hispanic/Latinx.
[c] Patients self-reporting as Asian, Hawaiian or Pacific Islander, American Indian or Alaskan Native, or multiracial were grouped together due to small percentages.

© 2024 Sun QW et al. *JAMA Network Open*.

eTable 2. Multivariate linear models for clinician FTE and individual quality metrics with interaction terms between clinician types

In eTable 2 and eTable 3, we performed sensitivity analyses using additional multivariate linear models with interaction effects between physicians, advanced-practice registered nurses, and other advanced practice providers intended to capture potential interdisciplinary dynamic effects, such as collaborative team-based care models and supervisory relationships. Primary exposure variables in eTable 2 includes raw FTE counts of physician, APRN, and PAs, and exposure variables in eTable 3 includes FTE of physician, APRN, and PAs per 1,000 visits per year. The sensitivity analysis generally supports the results of our main findings, suggesting that observed significant associations between clinician staffing levels and quality metrics are unlikely attributable to interdependencies among clinician types.

	b Coefficient (95% CI)		
Quality Metric, %	Physician FTEs	APRN FTEs	PA FTEs
Age-appropriate Childhood Vaccinations	0.54 (0.26, 0.81)***	0.24 (-0.05, 0.53)	0.96 (0.17, 1.75)
Cervical Cancer Screening	0.65 (0.44, 0.86)***	0.11 (-0.11, 0.33)	0.84 (0.23, 1.45)**
Breast Cancer Screening	0.48 (0.26, 0.70)***	0.06 (-0.17, 0.29)	0.43 (-0.19, 1.06)
Childhood and Adolescent BMI Measurement and Counseling	0.27 (-0.06, 0.60)	0.43 (0.09, 0.78)*	-0.66 (-1.61, 0.28)
Adult BMI Measurement and Appropriate Follow-up	-0.55 (-0.86, -0.23)***	0.55 (0.22, 0.88)***	-0.72 (-1.61, 0.17)
Tobacco Use Screening and Appropriate Intervention	-0.17 (-0.38, 0.03)	0.15 (-0.06, 0.36)	0.36 (-0.21, 0.94)
Appropriate Statin Therapy	0.11 (-0.02, 0.23)	0.10 (-0.03, 0.23)	0.10 (-0.25, 0.46)
Appropriate Aspirin or Antiplatelet Therapy	0.10 (-0.07, 0.27)	0.13 (-0.05, 0.31)	-0.01 (-0.50, 0.49)
Colorectal Cancer Screening	0.44 (0.22, 0.65)***	0.01 (-0.21, 0.24)	0.65 (0.04, 1.27)*
HIV Testing	0.91 (0.59, 1.23)***	0.17 (-0.17, 0.50)	-0.08 (-0.99, 0.83)
Depression Screening and Appropriate Follow-up	-0.08 (-0.35, 0.19)	0.31 (0.03, 0.59)*	-0.44 (-1.20, 0.33)
Depression in Remission	0.24 (0.01, 0.47)*	-0.17 (-0.41, 0.07)	-0.80 (-1.45, -0.15)*
Hypertension Controlled	0.06 (-0.07, 0.18)	0.10 (-0.04, 0.23)	0.41 (0.05, 0.78)*
Diabetes A1C Controlled	-0.00 (-0.13, 0.12)	0.04 (-0.10, 0.17)	-0.33 (-0.69, 0.03)

***p<0.001, **p<0.01, *p<0.05

© 2024 Sun QW et al. *JAMA Network Open*.

eTable 3. Multivariate linear models for clinician FTE per 1,000 visits and individual quality metrics with interaction terms between clinician types

	b Coefficient (95% CI)		
Quality Metric, %	Physician FTEs per 1,000 visits	APRN FTEs per 1,000 visits	PA FTEs per 1,000 visits
Age-appropriate Childhood Vaccinations	3.0 (-24.6, 30.6)	-28.4 (-46.2, -10.6)**	-48.5 (-85.3, -11.8)**
Cervical Cancer Screening	15.6 (-5.8, 37.0)	-19.0 (-32.8, -5.1)**	-32.9 (-61.5, -4.4)*
Breast Cancer Screening	19.7 (-2.3, 41.6)~	-12.2 (-26.4, 1.9)	-19.3 (-48.5, 10.0)
Childhood and Adolescent BMI Measurement and Counseling	12.3 (-19.8, 44.4)	-10.2 (-30.9, 10.5)	-25.8 (-68.5, 16.9)
Adult BMI Measurement and Appropriate Follow-up	-38.1 (-68.9, -7.3)*	-2.3 (-22.2, 17.6)	-6.7 (-47.8, 34.3)
Tobacco Use Screening and Appropriate Intervention	-4.6 (-24.6, 15.3)	-1.2 (-14.0, 11.7)	-0.7 (-27.2, 28.9)
Appropriate Statin Therapy	9.2 (-2.9, 21.3)	4.4 (-3.4, 12.2)	-6.4 (-22.5, 9.7)
Appropriate Aspirin or Antiplatelet Therapy	4.0 (-13.1, 21.0)	-2.4 (-13.4, 8.6)	1.9 (-20.7, 24.6)
Colorectal Cancer Screening	9.8 (-11.7, 31.4)	-13.1 (-27.0, 0.8)	-16.1 (-44.8, 12.6)
HIV Testing	45.8 (13.9, 77.6)**	-5.7 (-26.3, 14.8)	-47.7 (-90.2, -5.3)*
Depression Screening and Appropriate Follow-up	-2.7 (-29.4, 24.0)	3.1 (-14.1, 20.4)	0.5 (-35.0, 36.1)
Depression in Remission	42.8 (20.4, 65.1)***	12.5 (-1.9, 27.0)~	12.2 (-17.6, 41.9)
Hypertension Controlled	-14.5 (-27.1, -1.9)*	-6.9 (-15.0, 1.3)	-7.6 (-24.5, 9.2)
Diabetes A1C Controlled	10.4 (-2.0, 22.7)	1.7 (-6.2, 9.7)	-2.0 (-18.4, 14.5)

***p<0.001, **p<0.01, *p<0.05, ~p<0.10

© 2024 Sun QW et al. *JAMA Network Open.*

eFigure 1. Generalized additive models of physician FTE and rates of infant vaccinations

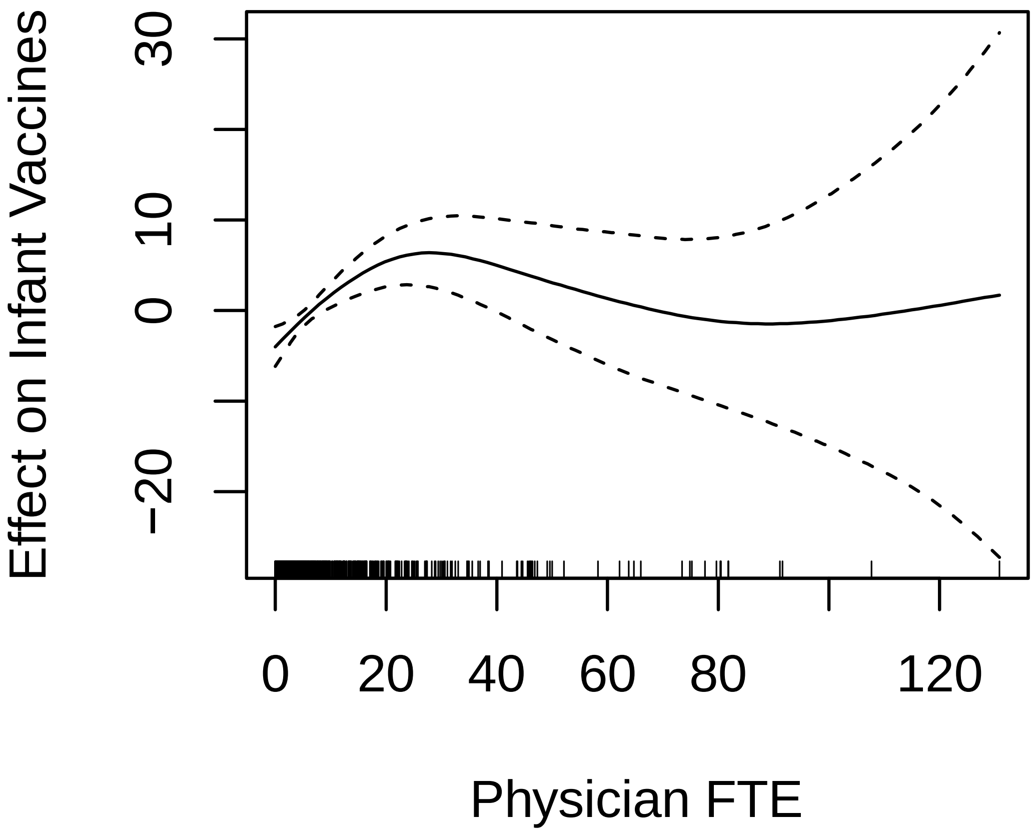

Y-axes quantify the estimated effect of the corresponding FTE on the specified quality metric. The solid line represents the smooth function, which approximates the actual shape of data rather than imposing a predetermined fit. Dotted lines represent the 95% confidence intervals. In eFigure 1, the minimum physician FTE count at which the association between physician FTE and rate of infant vaccines becomes positive is 9.25, determined by the point at which the curve and lower bound of the confidence interval is greater than 0. However, this association was not significant when adjusted for multiple comparisons (p=0.07)

© 2024 Sun QW et al. *JAMA Network Open*.

eFigure 2. Generalized additive models of PA FTE and rates of infant vaccinations

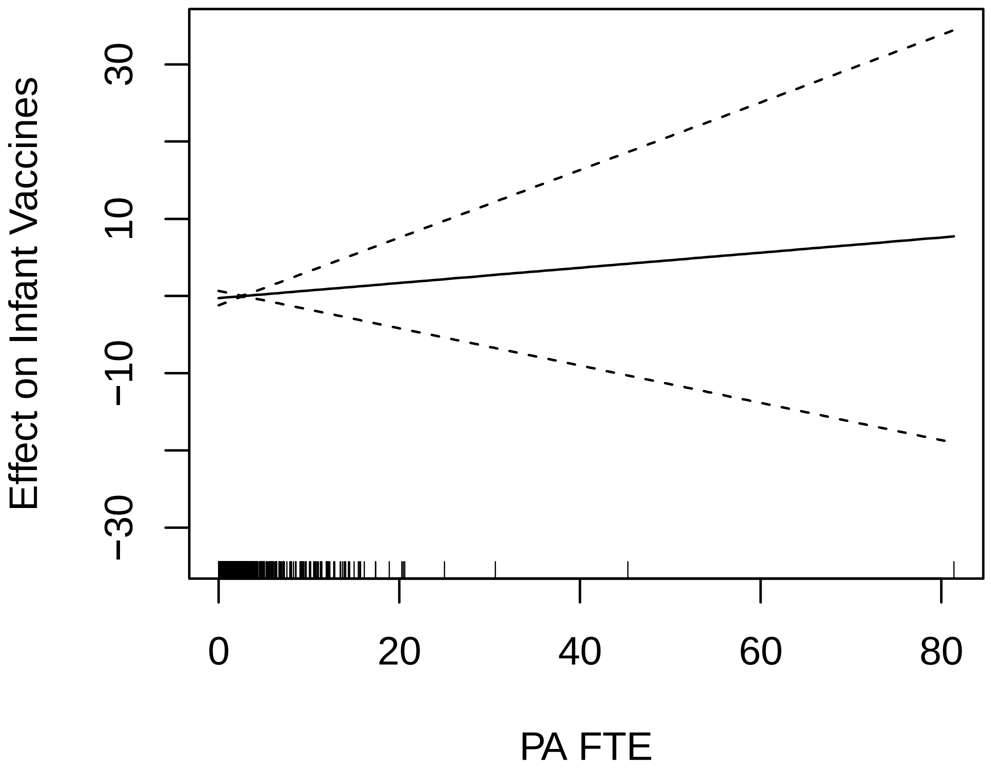

Y-axes quantify the estimated effect of the corresponding FTE on the specified quality metric. The solid line represents the smooth function, which approximates the actual shape of data rather than imposing a predetermined fit. Dotted lines represent the 95% confidence intervals. In eFigure 2, the association between PA FTE and rates of infant vaccinations is noted to be linear but not significant (p=0.56), as illustrated by the straight line. This signifies that there is no minimum PA FTE at which the association between PA FTEs and rates of infant vaccines becomes positive, and that GAMs are not most appropriate choice of model for this relationship.

© 2024 Sun QW et al. *JAMA Network Open*.

eFigure 3. Generalized additive models of physician FTE and rates of cervical cancer screening

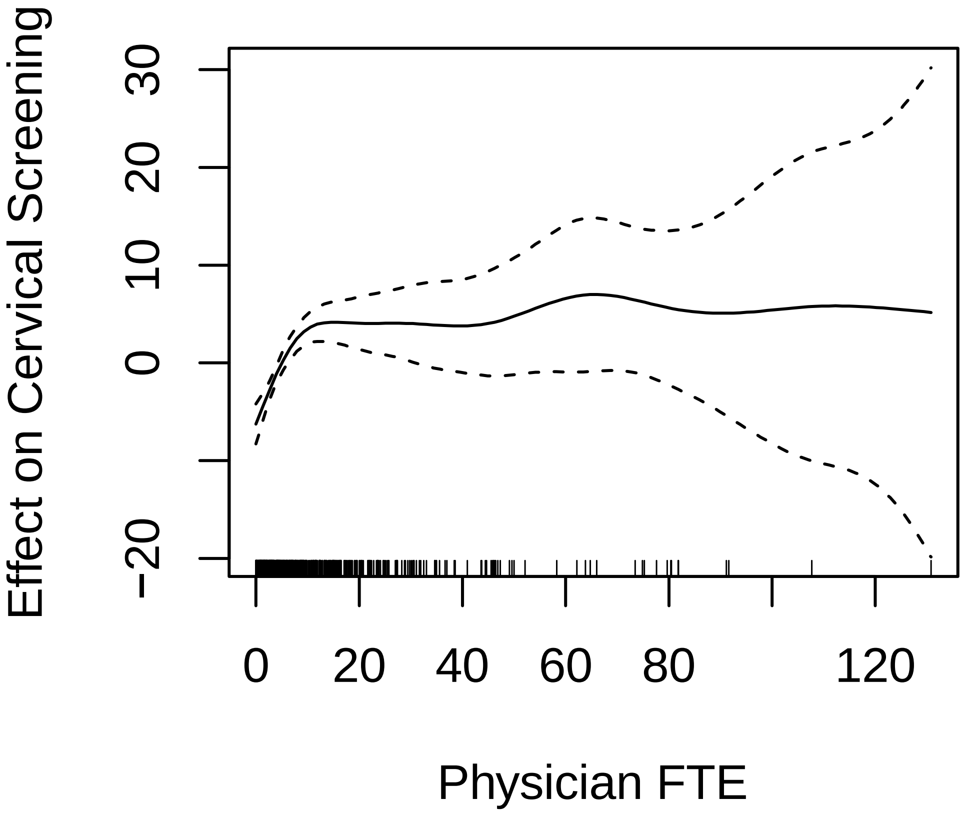

Y-axes quantify the estimated effect of the corresponding FTE on the specified quality metric. The solid line represents the smooth function, which approximates the actual shape of data rather than imposing a predetermined fit. Dotted lines represent the 95% confidence intervals. In eFigure 3, the minimum physician FTE count at which the association between physician FTE and rate of cervical cancer screening becomes positive is 6.61, determined by the point at which the curve and lower bound of the confidence interval is greater than 0 ($p=0.002$).

© 2024 Sun QW et al. *JAMA Network Open*.

eFigure 4. Generalized additive models of physician FTE and rates of breast cancer screening

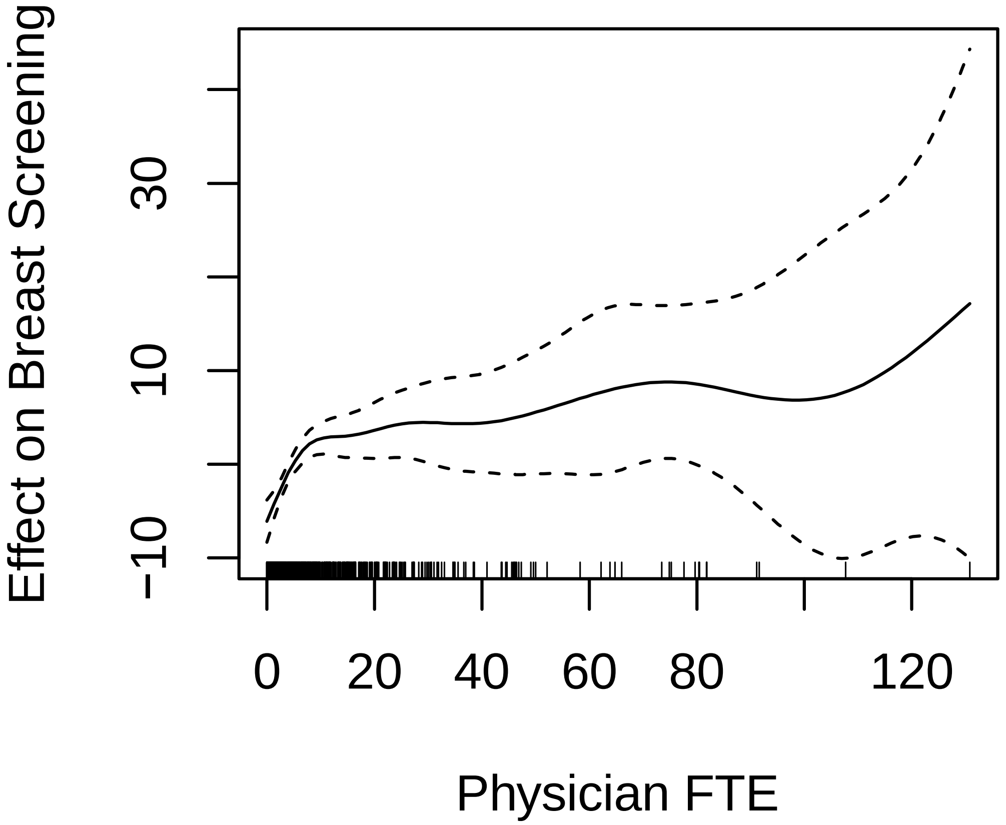

Y-axes quantify the estimated effect of the corresponding FTE on the specified quality metric. The solid line represents the smooth function, which approximates the actual shape of data rather than imposing a predetermined fit. Dotted lines represent the 95% confidence intervals. In eFigure 4, the minimum physician FTE count at which the association between physician FTE and rate of breast cancer screening becomes positive is 7.93, determined by the point at which the curve and lower bound of the confidence interval is greater than 0 (p=0.006).

© 2024 Sun QW et al. *JAMA Network Open*.

eFigure 5. Generalized additive models of physician FTE and rates of colorectal cancer screening

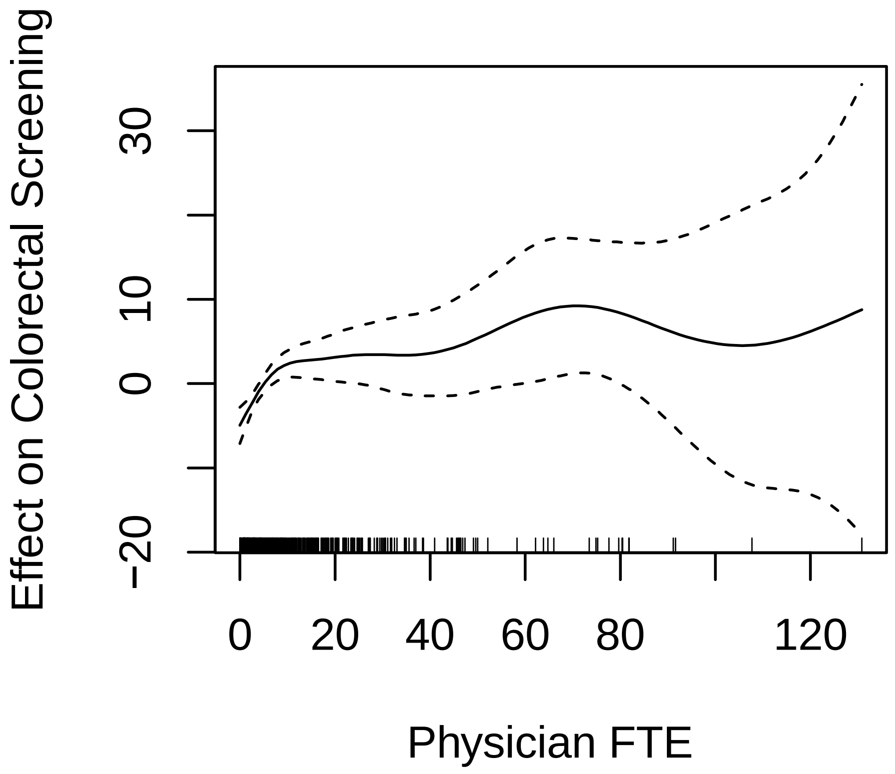

Y-axes quantify the estimated effect of the corresponding FTE on the specified quality metric. The solid line represents the smooth function, which approximates the actual shape of data rather than imposing a predetermined fit. Dotted lines represent the 95% confidence intervals. In eFigure 5, the minimum physician FTE count at which the association between physician FTE and rate of colorectal cancer screening becomes positive is 7.93, determined by the point at which the curve and lower bound of the confidence interval is greater than 0 ($p=0.02$).

© 2024 Sun QW et al. *JAMA Network Open*.

eFigure 6. Generalized additive models of APRN FTE and rates of adult BMI assessment and counseling

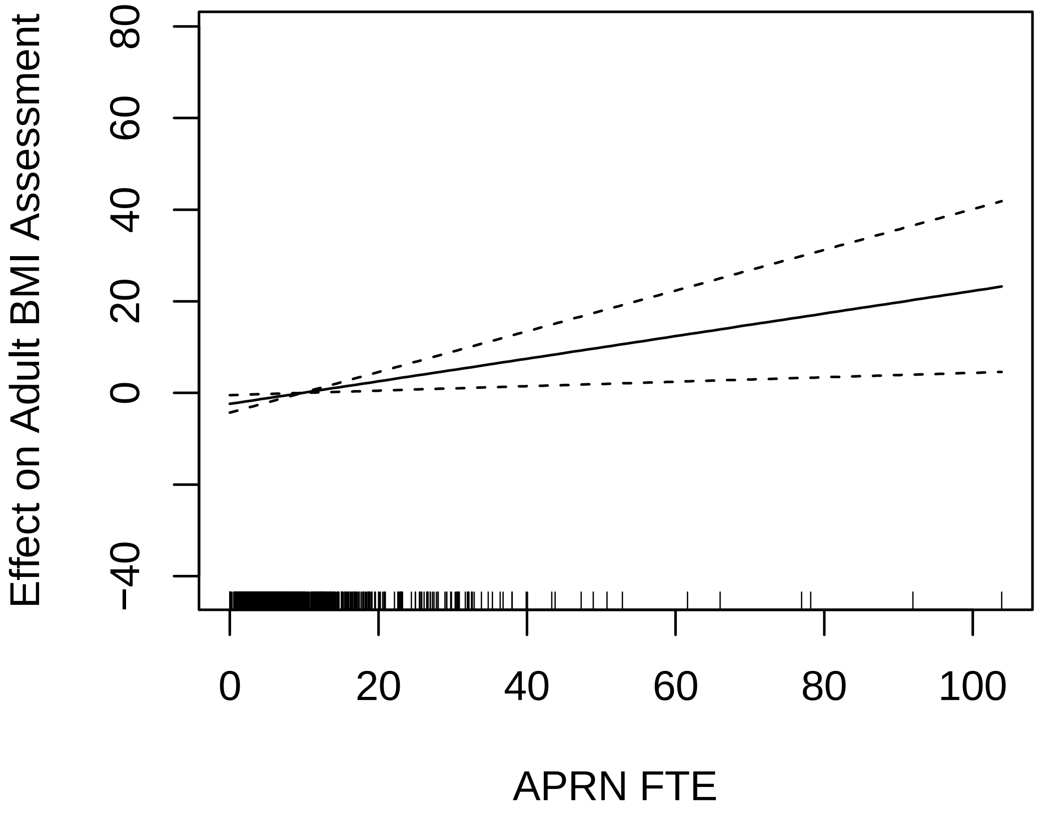

Y-axes quantify the estimated effect of the corresponding FTE on the specified quality metric. The solid line represents the smooth function, which approximates the actual shape of data rather than imposing a predetermined fit. Dotted lines represent the 95% confidence intervals. In eFigure 6, the association between APRN FTE and rates of adult BMI assessment and counseling is noted to be linear (p=0.04), as illustrated by the straight line. This signifies that there is no minimum APRN FTE at which the association between APRN FTEs and rates of adult BMI assessment becomes positive, and that GAMs are not most appropriate choice of model for this relationship.

© 2024 Sun QW et al. *JAMA Network Open*.

eFigure 7. Generalized additive models of physician FTE and rates of HIV testing

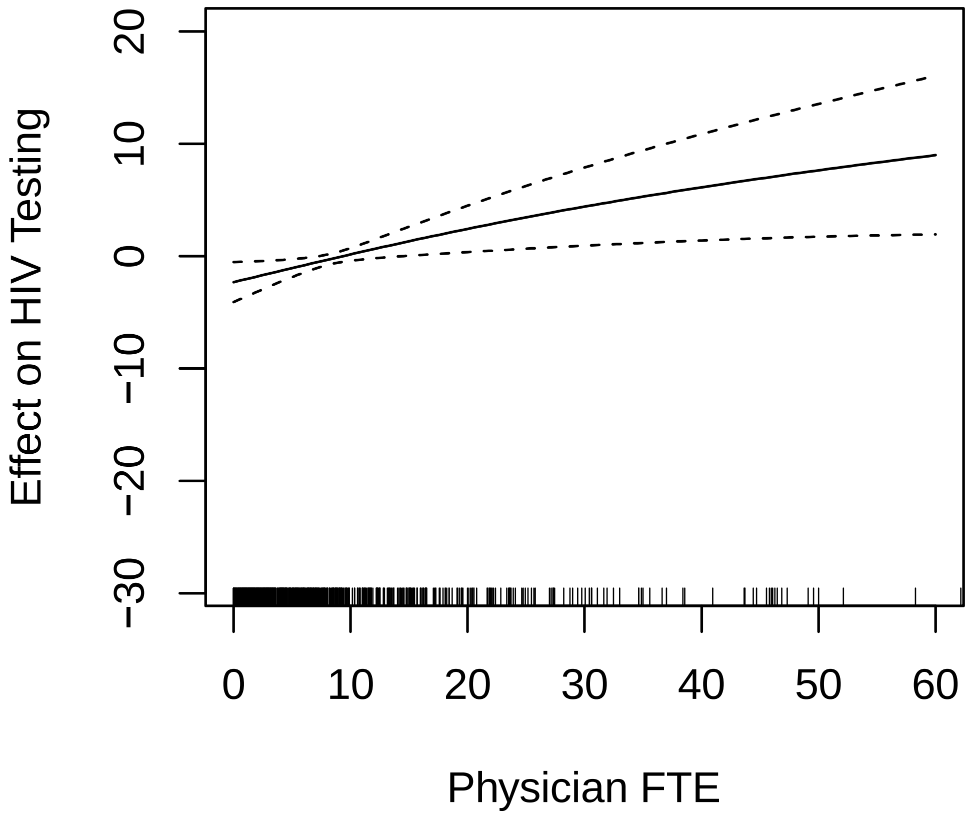

Y-axes quantify the estimated effect of the corresponding FTE on the specified quality metric. The solid line represents the smooth function, which approximates the actual shape of data rather than imposing a predetermined fit. Dotted lines represent the 95% confidence intervals. In eFigure 7, the minimum physician FTE count at which the association between physician FTE and rate of HIV testing becomes positive is 14.53, determined by the point at which the curve and lower bound of the confidence interval is greater than 0 (p=0.04).

© 2024 Sun QW et al. *JAMA Network Open*.

eFigure 8. Generalized additive models of APRN FTE and rates of HIV testing

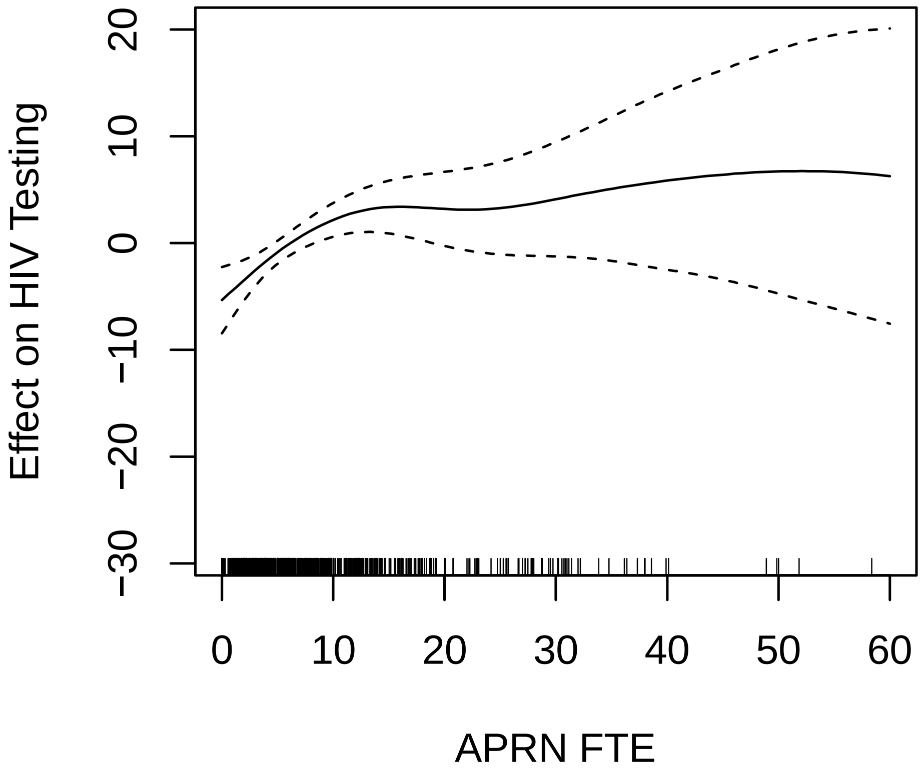

Y-axes quantify the estimated effect of the corresponding FTE on the specified quality metric. The solid line represents the smooth function, which approximates the actual shape of data rather than imposing a predetermined fit. Dotted lines represent the 95% confidence intervals. In eFigure 8, the minimum physician FTE count at which the association between physician FTE and rate of HIV testing becomes positive is 8.81, determined by the point at which the curve and lower bound of the confidence interval is greater than 0 (p=0.02).

© 2024 Sun QW et al. *JAMA Network Open*.

eFigure 9. Generalized additive models of physician FTE and rates of depression in remission

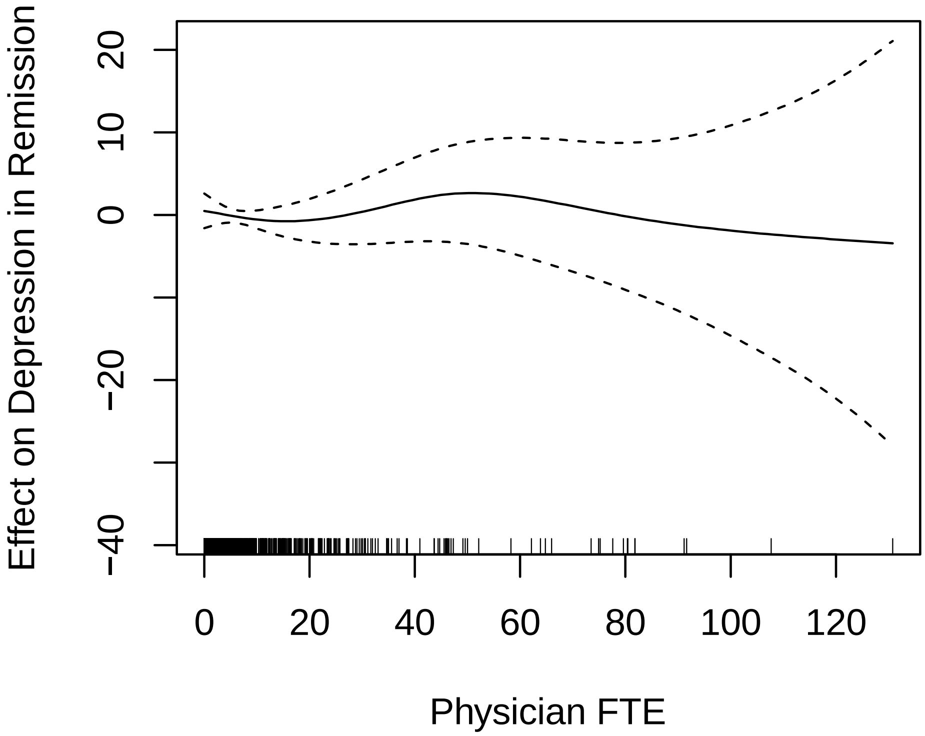

Y-axes quantify the estimated effect of the corresponding FTE on the specified quality metric. The solid line represents the smooth function, which approximates the actual shape of data rather than imposing a predetermined fit. Dotted lines represent the 95% confidence intervals. In eFigure 9, the nonlinear association between physician FTE counts and rate of depression in remission is nonsignificant (p=0.71), indicating there is no minimum FTE at which this association becomes positive.

© 2024 Sun QW et al. *JAMA Network Open*.
