# Supplementary material for: Clinician Staffing and Quality of Care in US Health Centers
Source: JAMA Netw Open. 2024 Oct 22;7(10):e2440140. doi: 10.1001/jamanetworkopen.2024.40140 (PMC11581487; doi:10.1001/jamanetworkopen.2024.40140)
Supplement: Supplement 2. — Data Sharing Statement [file jamanetwopen-e2440140-s002.pdf]

Data Sharing Statement

Sun. Clinician Staffing and Quality of Care in US Health Centers. *JAMA Netw Open*. Published October 22, 2024. doi:10.1001/jamanetworkopen.2024.40140

Data
Data available: Yes
Data types: Data (not involving human participants)
How to access data: https://data.hrsa.gov/tools/data-reporting/program-data/national
When available: With publication

Supporting Documents
Document types: None

Additional Information
Who can access the data: This data is publicly available.
Types of analyses: This data is publicly available.
Mechanisms of data availability: This data is publicly available.
